# GFP-Forked, a genetic reporter for studying *Drosophila* oocyte polarity

**DOI:** 10.1242/bio.039552

**Published:** 2018-12-31

**Authors:** Raju Baskar, Anna Bakrhat, Uri Abdu

**Affiliations:** Department of Life Sciences, Ben-Gurion University of the Negev, Be'er Sheva 8410501, Israel

**Keywords:** CRISPR, *Drosophila*, Forked, Oocyte, Polarity, Microtubules, NcMTOC

## Abstract

The polarized organization of the *Drosophila* oocyte can be visualized by examining the asymmetric localization of mRNAs, which is supported by networks of polarized microtubules (MTs). In this study, we used the gene *forked*, the putative *Drosophila* homologue of *espin*, to develop a unique genetic reporter for asymmetric oocyte organization. We generated a null allele of the *forked* gene using the CRISPR-Cas9 system and found that *forked* is not required for determining the axes of the *Drosophila* embryo. However, ectopic expression of a truncated form of GFP-Forked generated a distinct network of asymmetric Forked, which first accumulated at the oocyte posterior and was then restricted to the anterolateral region of the oocyte cortex in mid-oogenesis. This localization pattern resembled that reported for the polarized MTs network. Indeed, pharmacological and genetic manipulation of the polarized organization of the oocyte showed that the filamentous Forked network diffused throughout the entire cortical surface of the oocyte, as would be expected upon perturbation of oocyte polarization. Finally, we demonstrated that Forked associated with Short-stop and Patronin foci, which assemble non-centrosomal MT-organizing centers. Our results thus show that clear visualization of asymmetric GFP-Forked network localization can be used as a novel tool for studying oocyte polarity.

## Abstract (precis)

**Summary:** The novel asymmetric Forked network could be used as a genetic reporter for visualizing and studying oocyte polarity.

## INTRODUCTION

Correct localization of intracellular messenger RNAs (mRNAs) encoding morphogenetic proteins to their distinct subcellular domains are crucial for specification of the body axes of the *Drosophila* embryo. The roles of three major asymmetrically localized mRNAs, *gurken* (*grk*), *bicoid* (*bcd*) and *oskar* (*osk*), in the process of establishing axial patterning of the oocyte and embryo in mid-oogenesis were clearly established. *grk* is localized around the oocyte nucleus and determines the dorsal–ventral axis of the oocyte and embryo (Gonzalez-Reyes et al., 1995; Neuman-Silberberg and Schupbach, 1993, 1996; Roth et al., 1995), whereas *bcd* is localized to the extreme anterior of the oocyte and determines the anterior pattern of the embryo upon translation (Driever and Nusslein-Volhard, 1988a,b). At the same time, *osk* is localized to the posterior of the oocyte and initiates the development of future germ cells and the embryo abdomen (Ephrussi et al., 1991).

Asymmetric mRNA localization during mid-oogenesis depends on microtubules (MTs), actin networks and motor proteins. During mid-oogenesis (i.e. stages 9 and 10), MTs within the oocyte are organized asymmetrically, with non-centrosomal MT-organization centers (ncMTOCs) being localized solely to the anterior and lateral cortexes of the oocyte (Huynh and St Johnston, 2004; Nashchekin et al., 2016; Theurkauf, 1994). Regulation of the asymmetrical oocyte MT network is mainly controlled by two sequential processes. Initially, an MTOC positioned at the posterior end of the oocyte, close to the nucleus, which is in a symmetric position at this stage, is established. Signaling from *grk* to EGF receptors at the posterior end of the oocyte (stage 6–7) establishes posterior follicle cell fate (Gonzalez-Reyes et al., 1995; Roth et al., 1995). Subsequently, an unknown signal produced by posterior follicle cells leads to the establishment of Par-1 kinase activity in the posterior oocyte cortex, thereby defining the antero-lateral versus posterior cortical domain in the oocyte (Doerflinger et al., 2006; Shulman et al., 2000). In addition, the same unknown signal triggers disassembly of the posterior MTOC. At the same time, nucleation of new MTs at the oocyte anterior end results in a reversal of polarity within the oocyte (Gonzalez-Reyes et al., 1995; Roth et al., 1995; Theurkauf et al., 1992). Par-1 kinase now restricts the polarization of MTs to the antero-lateral region of the oocyte by suppressing MT nucleation at the oocyte posterior end (Doerflinger et al., 2006; Parton et al., 2011).

Visualization of the asymmetric organization of the *Drosophila* oocyte is achieved either by *in situ* hybridization or by staining with antibodies directed against several localized mRNA, such as *grk*, *bcd* and *osk*. In addition, the polarized organization of oocyte MTs can be demonstrated either by staining using anti-tubulin antibodies or by the expression of MT-associated proteins fused to GFP (Parton et al., 2011). Detection of the polarized MT network using anti-tubulin antibodies, however, requires the use of a special protocol (Legent et al., 2015).

In studying the role of *forked* gene, the *Drosophila* homologue of *espin*, in oogenesis, we discovered that ectopic expression of GFP-Forked protein could be used as a novel tool for analyzing oocyte polarity. First, we demonstrated that oocytes containing mutations in *forked* showed no defects in polarity. On the other hand, upon over-expression of GFP-Forked, the protein first accumulated at the oocyte posterior end and was then restricted to the anterolateral region of the oocyte cortex in mid-oogenesis. We showed that this unique asymmetric localization of GFP-Forked depends on the polarized MT network. We further found that ectopic expression of Forked is associated with Short-stop and Patronin foci. Thus, our results reveal that the novel asymmetric Forked network can be used as a genetic reporter for visualizing and studying oocyte polarity.

## RESULTS

### Forked is not required for oocyte axis determination

Previous work from our lab and others revealed similarities between oocyte and bristle cytoskeleton organization (Abdu et al., 2006; Amsalem et al., 2013; Bitan et al., 2010; Dubin-Bar et al., 2011, 2008; Lundh et al., 2011; Otani et al., 2015; Shapiro and Anderson, 2006). It was shown that the complex of proteins containing Spn-F, Ik2 and Jvl plays a similar role in both oocyte and bristle actin network organization. Generating actin bristle bundles requires two actin-bundling proteins, Singed (sn), the *Drosophila* Fascin homologue, and Forked, the putative *Drosophila* homologue of *espin*. Previously, it was shown that Sn is required for the formation of cytoplasmic actin bundles in nurse cells (Cant et al., 1994). However, the role of *forked* in oogenesis is still unknown.

**Fig. 1.**
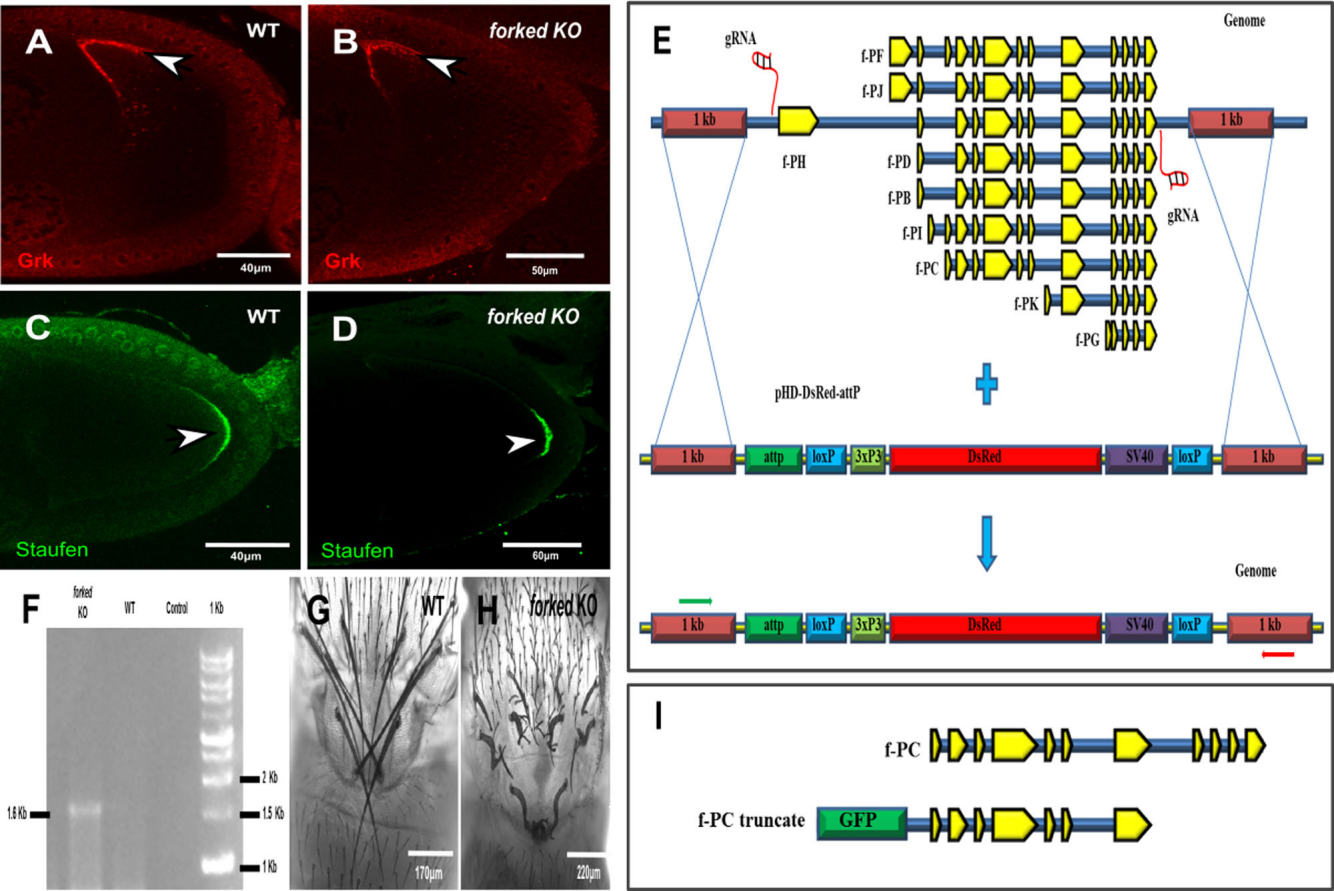
**Forked is not required for oocyte axis determination.** Confocal images of a representative stage 9 egg chamber from WT (wild type; A,C) and CRISPR/Cas9-generated *forked* knockout (KO) flies (B,D) stained with anti-Grk (red; A,B) and anti-Staufen antibodies (green; C,D). Grk, which is responsible for determination of the dorsal–ventral axis of the embryo, is localized to the dorsoanterior corner of the oocyte both in WT (arrowhead in A) and in *forked* KO (arrowhead in B) egg chambers. For analysis of defects in the posterior axis of the oocyte, Staufen, which is required for the localization of the posterior axis determinant Oskar, was used. Staufen is localized to the posterior end of the oocyte both in WT (arrowhead in C) and *forked* KO (arrowhead in D) egg chambers. (E) Schematic diagram showing the nature of *forked* loci and generation of *forked* KO flies (null allele) using CRISPR-mediated homology-directed repair (HDR) with the donor pHD-DsRed-attP vector. All nine *forked* isoform exons are shown in yellow on loci, shown in blue. Green and red arrows represent the binding sites of the forward and reverse primers used for genotyping, respectively. (F) PCR analysis of the genotyping of *forked* KO flies. The 1.6 kb band in the *forked* KO lane depicts the complete deletion of *forked* from the genome and its replacement with DsRed using the primers depicted in (E) by green and red arrows. Confocal images of the thorax region from the pharate adult of WT (G) and *forked* KO (H) flies. (I) Graphical illustration showing construction of a truncated form of GFP-tagged Forked isoform C.

Since the *f^36a^* (Hoover et al., 1993) allele failed to eliminate all *forked* splice forms and there is no molecular characterization for the *f^15^* allele containing a stop codon at Q206 (flybase; FBrf0191634), we decided to delete the *forked* gene using CRISPR/Cas9 technology. For this, we generated the null allele so as to eliminate all alternative splice forms of *forked* (Fig. 1E). Indeed, our PCR analysis revealed that we replaced the entire *forked* genomic region with dsRed (Fig. 1F). Moreover, homozygous alleles showed aberrant *forked* bristle phenotype (Fig. 1H). Moreover, we found that *forked* null allele females were fertile (Table S1)*.*

Next, we tested whether *forked* plays a role in oocyte polarity. As a read-out for any defect in oocyte polarity, we tested the localization of Gurken (Grk), which is responsible for determination of the dorsal-ventral axis of the egg (Gonzalez-Reyes et al., 1995), and Staufen (Stau), which is required for the localization of both the anterior axis determinants Bicoid (Bcd) and the posterior axis determinant Oskar (Osk) (St Johnston et al., 1991). We found that *forked*-deleted flies presented no obvious defects in terms of the localization of either Grk or Stau (Fig. 1B,D; *n*=20, 100%).

### Forked marks a distinct asymmetric network in the oocyte

**Fig. 2.**
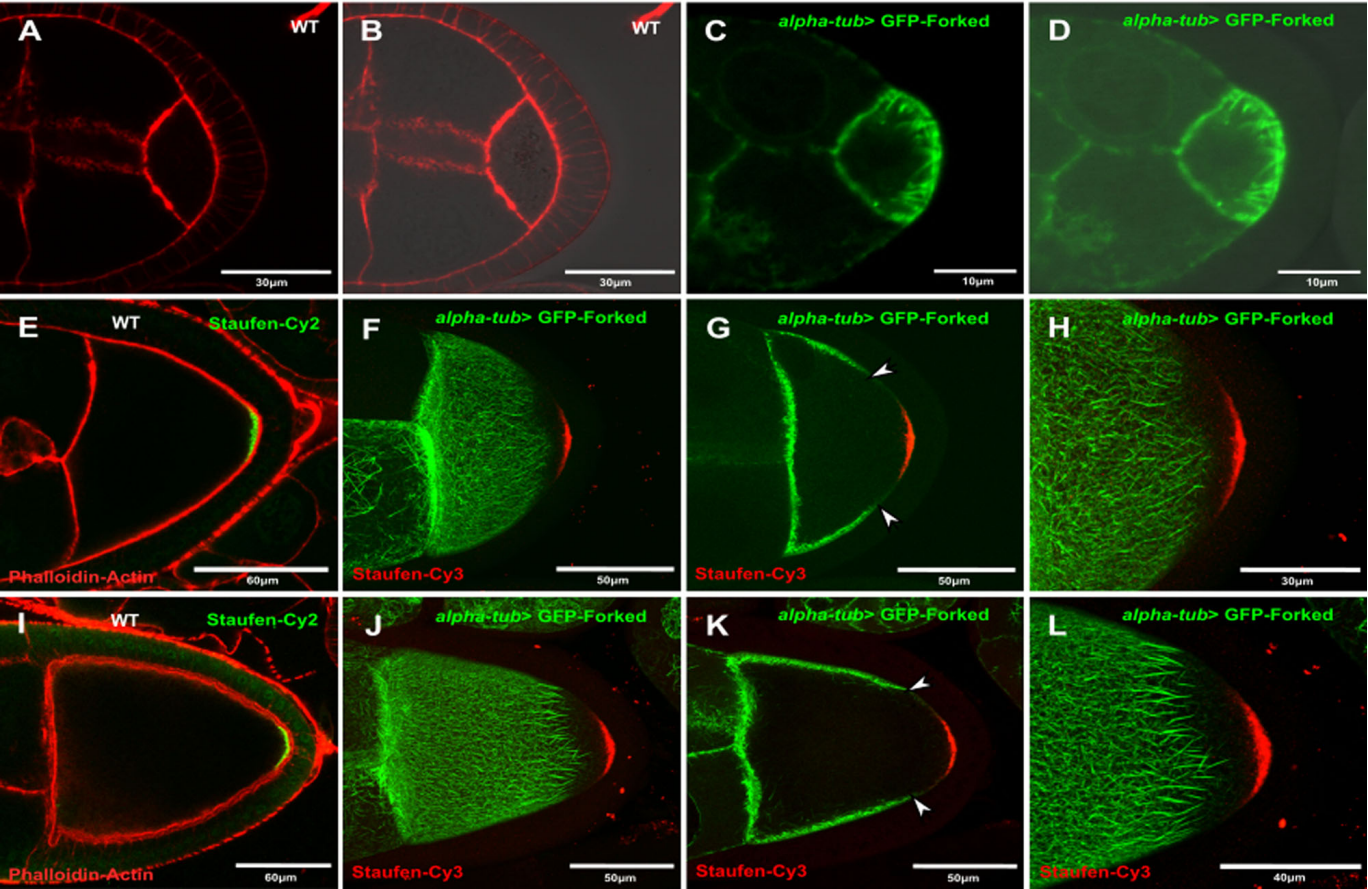
**Forked marks a distinct asymmetric network in oocytes.** Confocal images from (A,B) WT stage 5 egg chambers stained with phalloidin (red; A); B is the image shown in A seen with DIC. (C,D) *alpha-tub>* GFP-Forked stage 5; D is the merged image of C seen with DIC. WT stage 9 (E) and stage 10 (I) egg chambers stained with phalloidin (red; E,I) and anti-Staufen antibodies (green; E,I). *alpha-tub>* GFP-Forked stage 9 (F–H) and stage 10 (J–L) egg chambers were stained with anti-Cy3-conjgated anti-Staufen antibodies. Images (F,J) and (H,L) are confocal Z-series projections. (G) and (K) show single confocal slices from the Z-series projections of images (F) and (J), respectively. Arrowheads in (G) and (K) point towards the limit of the asymmetric network marked by GFP-Forked.

**Fig. 3.**
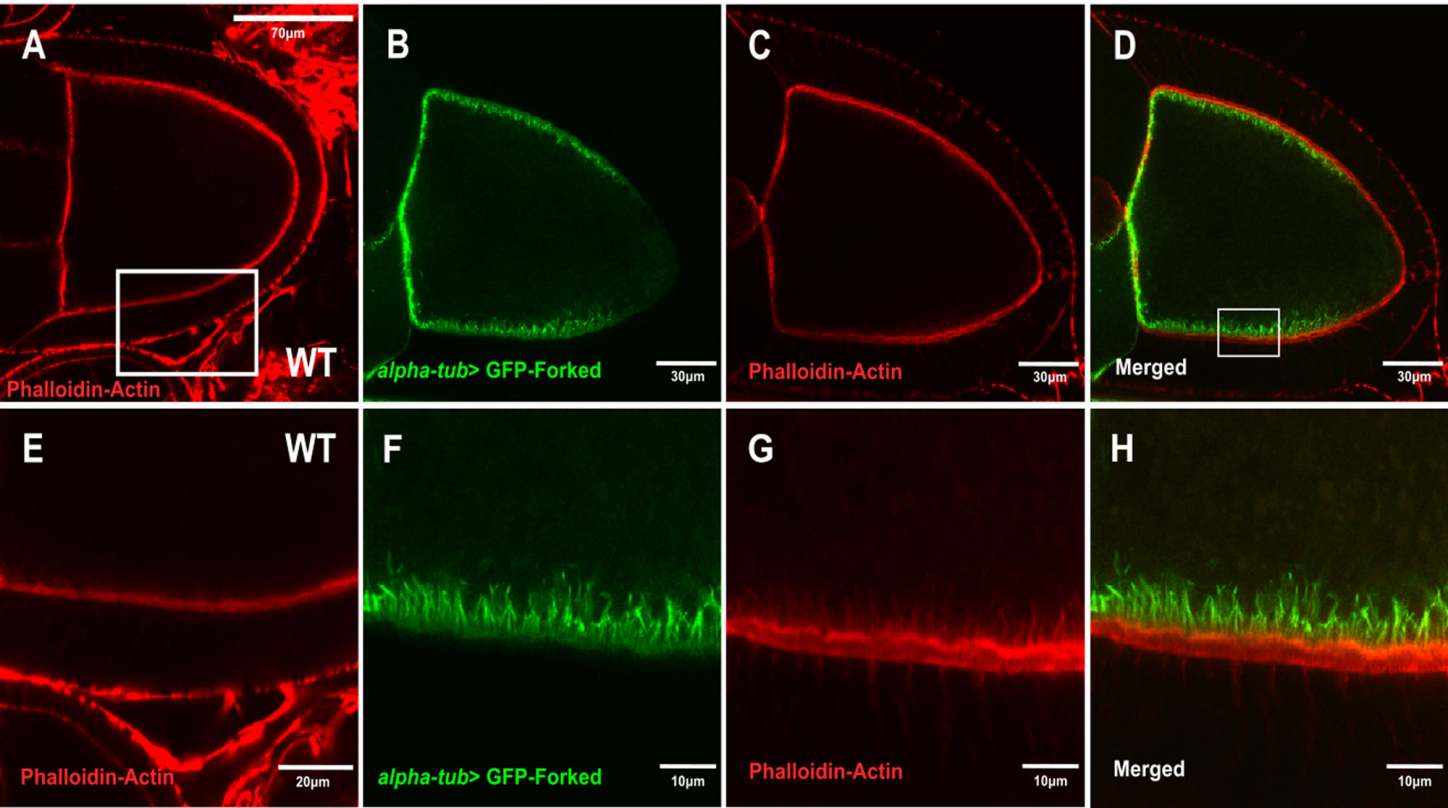
**Colocalization of the asymmetric Forked network with an actin marker.** (A,E) Confocal images of WT stage 10 egg chambers stained with phalloidin for actin (red). (E) Enlargement of the boxed region of an antero-lateral segment from (A). (B–D,F–H) *alpha-tub>* GFP-Forked stage 10 egg chambers stained with phalloidin for actin (red). (F–H) Enlargement of the boxed region of an antero-lateral segment from (D).

Although our results showed that Forked is not required for oocyte polarity, we tested the localization pattern of ectopic GFP-Forked expression. Since it had been shown that extra copies of the *forked* gene affected bristle development (Petersen et al., 1994), we generated a truncated form of the *forked* gene isoform C fused to DNA encoding GFP (Fig. 1I (see Material and Methods). We found that although the GFP-Forked chimera was localized to bristle actin bundles (Fig. S1A–C), it failed to rescue the *forked* bristle phenotype (Fig. S1E–F). Next, when we analyzed the localization of GFP-Forked in the egg chamber. To our surprise, we found that the ectopic expression of GFP-Forked presented a unique localization pattern. At stage 5, GFP tagged-Forked decorated a filamentous network that radiated from the circumferential cortical posterior surface of the oocyte towards the oocyte cytoplasm (Fig. 2C,D). However, in stage 9 egg chambers, this localization pattern had changed, such that the GFP tagged-Forked filamentous network was now restricted to the anterolateral cortex of the oocyte (Fig. 2F–H) and was completely absent at the posterior end of the oocyte (Fig. 2H). Moreover, at stage 10, this asymmetrical localization pattern of GFP-Forked could still be detected (Fig. 2J–L). We tested for effects of GFP-Forked expression and found that overexpression of GFP-Forked had no effect on female fertility (Table S2). We then asked whether this asymmetric filamentous network colocalized with actin. Using the general actin marker phalloidin, we found that the filamentous asymmetric network decorated by Forked indeed colocalized with actin (Fig. 3B–D,F–H). To summarize, our results suggest that ectopic expression of Forked generates a distinct filamentous network in the oocyte.

### The asymmetric Forked network depends on MTs

**Fig. 4.**
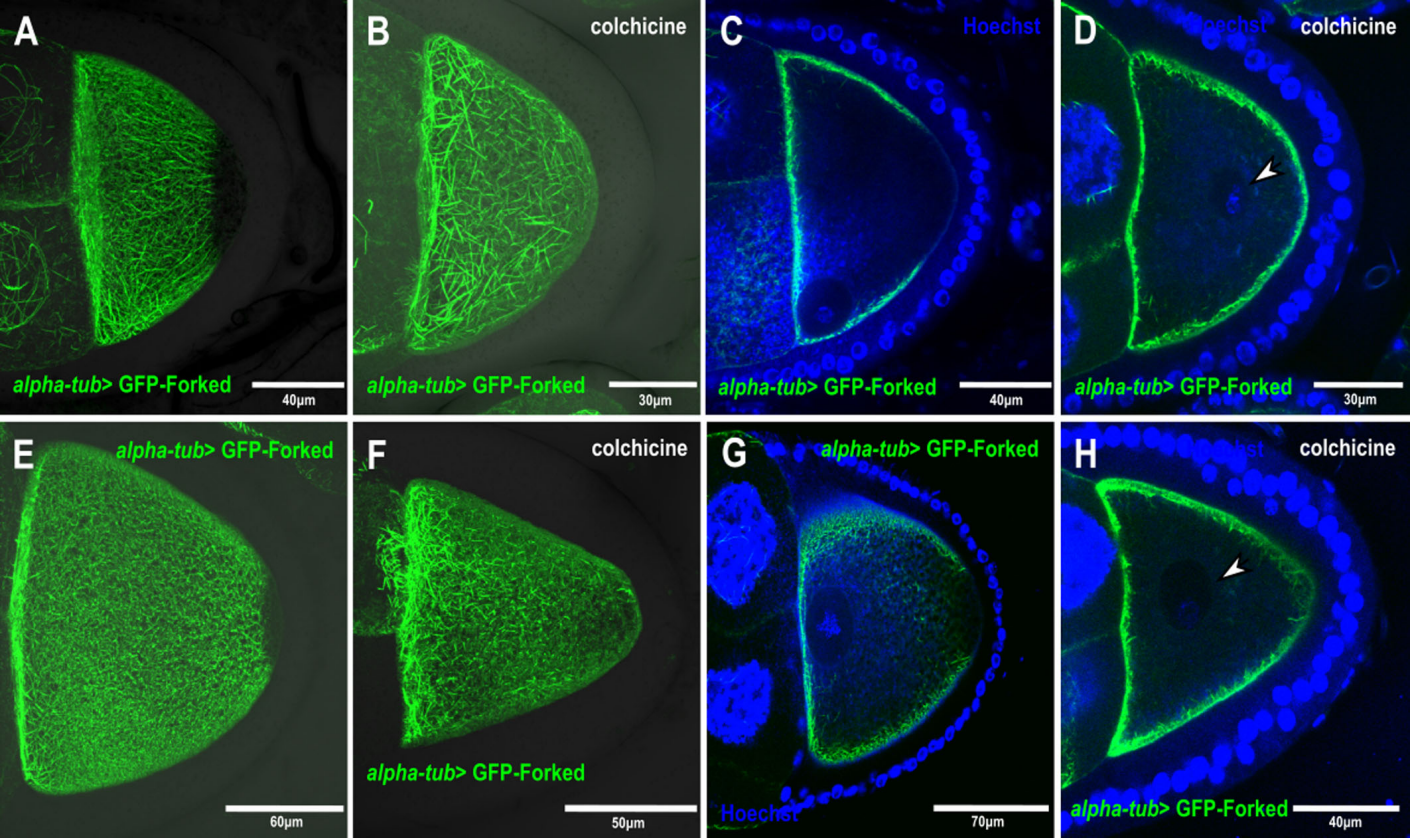
**The asymmetric Forked network depends on MTs.** (A–D) Confocal images from stage 9 and stage 10 (E–H) egg chambers expressing *alpha-tub>* GFP-Forked, flies fed with colchicine (B,D,F,H) and stained with Hoechst stain to view the nucleus (blue) (C,D,G,H). (A,B) and (E,F) are confocal Z-series projections, (C,D) and (G,H) are single confocal slices from the Z-series projections of images (A,B) and (E,F), which was merged with the Hoechst-stained image (blue), respectively. Arrowheads in (D) and (H) mark the oocyte nucleus.

We asked whether this decorated filamentous and asymmetric Forked network depended on MTs. For this purpose, flies expressing GFP-tagged Forked in the germline were fed with the MT-depolymerizing agent colchicine. We found that feeding flies with colchicine affected MT organization, as reflected by the mislocalization of the oocyte nucleus (Fig. 4D,H). Moreover, the decorated filamentous Forked network was no longer organized in an asymmetrical manner in the egg chambers, instead being diffusely distributed throughout the oocyte cortical surface (*n*=40, 100%; Fig. 4B,D,F,H). This indicates that the filamentous asymmetric Forked network is dependent on MT organization.

### The asymmetric Forked network depends on a polarized MT network

**Fig. 5.**
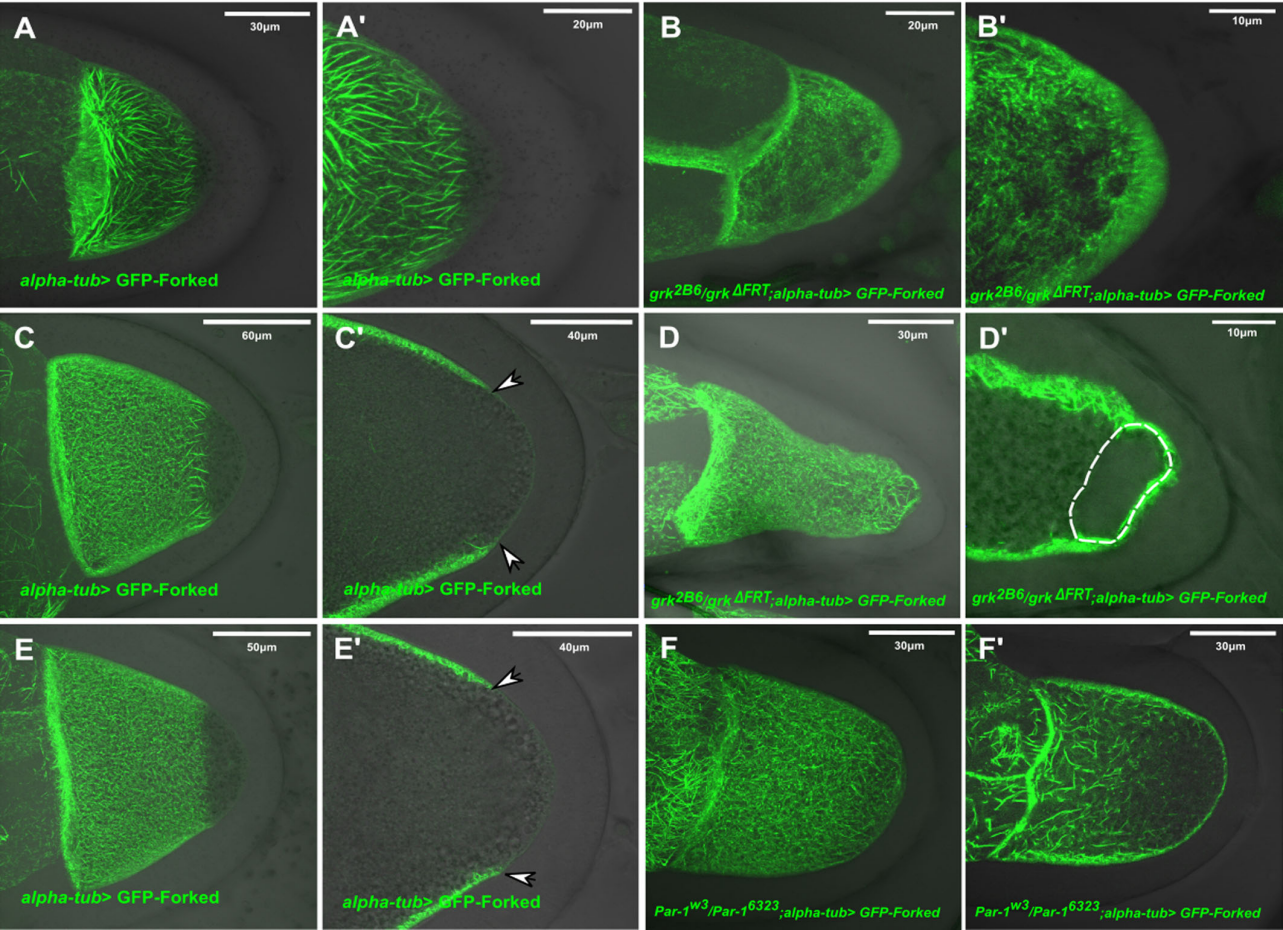
**The asymmetric Forked network depends on a polarized MT network.** Confocal Z-series projections of stage 9 egg chambers from WT (A,A′) and *grk* mutant flies (B,B′) expressing *alpha-tub>* GFP-Forked, merged with DIC images. Confocal Z-series projections of stage 10 egg chambers from WT (C) and *grk* mutant flies (D) expressing *alpha-tub>* GFP-Forked, merged with DIC images. C′ and D′ are single confocal slices from the Z-series projections of images C and D, merged with DIC images. Confocal Z-series projections of stage 10 egg chambers from WT (E) and *par-1* mutant flies (F) expressing *alpha-tub>* GFP-Forked, merged with DIC images. E′ and F′ are single confocal slices from the Z-series projections of image E and F, merged with DIC images. Arrowheads in (C′) and (E′) point towards the limit of the asymmetric network marked by GFP-Forked.

Next, we tested whether the novel asymmetrical Forked network requires a polarized MT network. We therefore first analyzed the localization pattern of GFP-tagged Forked in a *grk* mutant background, where during mid-oogenesis the MTs fail to repolarize and the oocyte nucleus often fails to migrate. We found that in both stage 9 and 10 egg chambers from *grk* mutants (*n*=45, 100%), the Forked filamentous network was diffusely localized throughout the cortical surface of the entire oocyte (Fig. 5B,B′,D,D′) and was no longer restricted to the anterolateral cortex. Moreover, at stage 10 (*n*=20, 25%; Fig. 5C), the filamentous Forked network generated a cage-like structure around the mislocalized oocyte nucleus (Fig. 5C′).

Since it has been reported that PAR-1 is required for polarized MT organization in the oocyte by preventing MT nucleation sites from forming at the posterior cortex (Parton et al., 2011), we analyzed the localization pattern of GFP-tagged Forked in a *par1* hypomorph mutant background that allows egg chambers to progress to mid-oogenesis (Parton et al., 2011; Shulman et al., 2000). In stage 9–10 egg chambers of *par1* mutant females, the filamentous Forked network was no longer organized in an asymmetrical manner, instead being diffusely distributed throughout the entire cortical surface of the oocyte (Fig. 5F,F′, *n*=35, 100%).

Another factor required for organizing the polarized MT network in the oocyte is the actin-MT cross linker *short stop* (*shot*). Recently, it was shown that *shot*, along with *patronin*, a *Drosophila* MT minus-end-binding protein, which encodes a CAMSAP homologue, are both required for MT organization in the oocyte by assembling non-centrosomal MTOCs at the antero-lateral cortex of the oocyte (Nashchekin et al., 2016). Since *shot* mutant egg chambers fail to develop past earlier stage of oogenesis, we used RNAi expressed in the germline via the UAS GAL4 system to downregulate levels of the *shot* transcript. We found in *shot* knockdown egg chambers, the filamentous Forked asymmetric network was also diffusely distributed throughout the cortical surface of the oocyte (*n*=20, 100%; Fig. S2C,D). In summary, we observed that whenever the polarized MT network was disrupted, the filamentous Forked network also lost polarity. Therefore, the asymmetric Forked network depends on a polarized MT network.

### Forked is associated with ncMTOCs

**Fig. 6.**
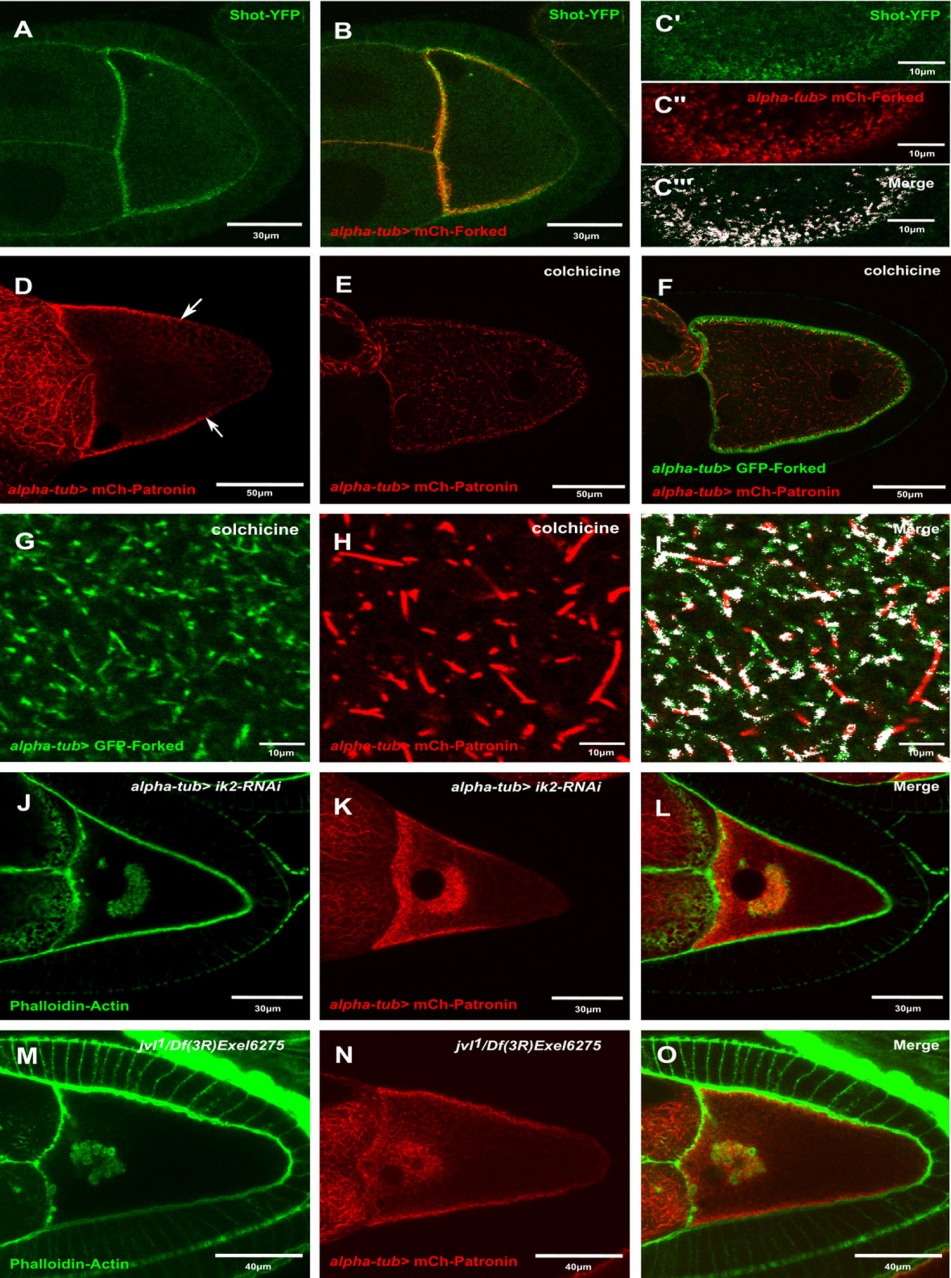
**Forked is associated with ncMTOCs.** Confocal image of stage 9 egg chambers expressing both (A) Shot-YFP, (B) *alpha-tub>* mCherry-Forked and (C′–C′′′) rectangle slices from the cortex region of egg chamber expressing both (C′) Shot-YFP and (C″) *alpha-tub>* mCherry-Forked. (C′′′) The colocalization pixel map of the merged image showing the region of colocalization regio in white. (D) Confocal image of stage 10 egg chambers expressing *alpha-tub>* mCherry-Patronin. (E–I) Confocal images of stage 10 egg chambers expressing (F) both *alpha-tub>* mCherry-Patronin (E) and *alpha-tub>* GFP-Forked from flies fed with colchicine. Confocal images of the cortex region from the stage 10 (G–I) egg chambers (D–F). (I) The colocalization pixel map of the merged image showing the region of colocalization in white. (J–L) Confocal images of stage 10 egg chambers from flies expressing both (J) *Ik2 RNAi* and (K) *alpha-tub>* mCherry-Patronin, stained with Phalloidin for actin (green) (J). (M–O) Confocal images of stage 10 egg chambers from *jvl* mutant flies expressing (N) *alpha-tub>* mCherry-Patronin, stained with Phalloidin for actin (green) (M). (O) Merged image. Arrows in D point towards the limit of the asymmetrical microtubule network marked by mCherry-Patronin.

Recently, it was shown that the ncMTOCs, which comprises Shot and Patronin, localizes to the anterolateral region of the oocyte (Nashchekin et al., 2016). Since the asymmetric localization pattern of Forked resembles that of Shot and Patronin, we assessed whether the ectopic expression of Forked generated this asymmetric network with ncMTOCs. First, we checked whether this asymmetric network colocalized with Shot. Using the UAS-Gal4 system, we expressed mCherry-Forked in the background of flies endogenously expressing Shot-YFP. We found that Shot-YFP colocalized with the Forked-asymmetric network (Fig. 6A–C‴). Closer examination revealed that Forked was associated with Shot foci along the antero-lateral cortex. In addition, we analyzed the colocalization of Shot-YFP with mCherry-Forked using the Fiji ImageJ software colocalization plug-in. We found that 95% of Shot-YFP colocalized with mCherry-Forked. Then, we considered whether disruption of the polarized MT network with colchicine was responsible for the change in Forked localization. Accordingly, flies expressing both mCherry-Patronin and GFP-Forked in the germline were fed with colchicine. We found that similar to the diffuse pattern of Forked (Fig. 6E–F) seen upon such drug treatment, Patronin foci were diffusely distributed throughout the oocyte, when compared to untreated animals (*n*=30, 100%; Fig. 6D), and colocalized with Forked (Fig. 6G–I). Furthermore, using the Fiji ImageJ software colocalization plug-in, we found that 82.5% of patronin foci colocalized with Forked. Since we found that the asymmetric Forked network associated with ncMTOCs, we asked whether ncMTOCs is also associated with actin-enriched aggregates in mutants with abnormal actin networks, such as *ik2, spn-F* and *jvl* mutants (Abdu et al., 2006; Dubin-Bar et al., 2011; Shapiro and Anderson, 2006). We found that mCherry-Patronin was associated with ectopic actin structures in both *ik2*-RNAi (Fig. 6J–L) and *jvl* mutants (Fig. 6M–O). Thus, these results suggest that the ectopic expression of Forked in the ovary generates an asymmetric network, which emanates from ncMTOCs in the oocyte.

### Forked can be used as a marker for visualizing the polarized oocyte MT network

**Fig. 7.**
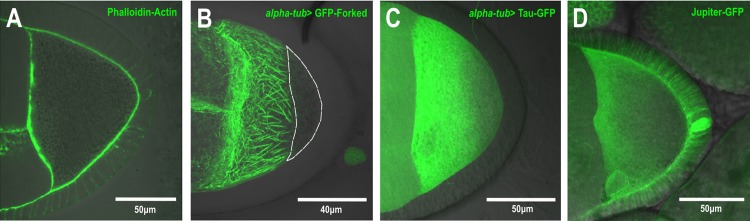
**Forked can be used as a marker for visualizing oocyte polarized MT networks.** Confocal image of stage 9 egg chambers from WT flies (A) stained with phalloidin for actin (green) merged with a DIC image. (B–D) Confocal Z-series projections of stage 9 egg chambers from WT flies expressing *alpha-tub>* GFP-Forked (B), *alpha-tub>* GFP-Tau and GFP-Jupiter (D), merged with DIC images. White dotted line represents the clear border of the posterior region (B).

We found that the asymmetric Forked network depends on polarized MTs and is associated with ncMTOC, suggesting that GFP-Forked could be used as a marker for studying oocyte polarity. Previously, several MT-associated proteins (MAPs) fused to fluorescent markers were used to study polarized MTs in oocytes (Parton et al., 2011, 2011). To demonstrate the strength of our proposed marker, we compared its localization pattern with that of two known MT markers, Tau and Jupiter. All three markers showed a decreasing gradient in the anterior to posterior direction (Fig. 7B–D). However, whereas in the case of Tau-GFP (Fig. 7C) and Jupiter-GFP (Fig. 7D), this gradient ended at the oocyte posterior region. In the case of GFP-Forked (Fig. 7B), the protein was completely absent from this region, thus generating a clear border of the posterior region. In summary, these findings reveal that GFP-Forked can be used as an additional marker for studying oocyte polarity.

## DISCUSSION

We previously found that Spn-F, together with IK2, plays a role in both oocyte polarity maintenance (Amsalem et al., 2013; Dubin-Bar et al., 2008) and bristle development (Otani et al., 2015). We therefore wanted to test whether other factors important for bristle formation also assume a role in oogenesis. Forked is an actin cross-linker protein that is required for the formation of actin bundles during bristle development (Petersen et al., 1994). We found that *forked* knockout females were fertile and showed no obvious defects in the localization of either Grk or Stau (Fig. 1B,D). These results demonstrate that the *forked* gene is not required for oocyte development.

Oocyte polarity in *Drosophila* is established in several steps, which involve dynamic changes in the MT network. Initially, centrosomes migrate from nurse cells towards the oocyte, accumulating at the posterior end of the oocyte nucleus, where a new MT-organizing center forms. All MTs are nucleated at this posterior MT-organizing center in stage 1 to stage 6 egg chambers (Bolivar et al., 2001; Mahowald and Strassheim, 1970). During the same stages, the asymmetric Forked network that we described here also assembles at the posterior end of the oocyte. In egg chambers at around stage 6/7, an as yet unknown signal from the posterior follicle cells serves to disassemble this posterior MTOC in response to Gurken to EGFR signaling. The formation of new MTs is subsequently initiated at the anterior and lateral cortexes, leading to a reversal of MT polarity in the oocyte. This new polarity, whereby MT minus-ends are anchored at the anterior and lateral cortexes, is crucial for the localization of axis determinants (Theurkauf et al., 1992). Significantly, the asymmetric Forked network that we describe here also underwent a posterior to anterior transition. We have shown that the asymmetric distribution of this Forked network depends on MTs, using *gurken* mutants, *par-1* mutants, and colchicine treatment. Indeed, we found that Forked colocalized with both Shot and Patronin foci, and that abnormal ectopic actin clogs are associated with ncMTOCs in the oocyte. Thus, the finding that Forked is associated with ncMTOCs explains the sensitivity of the asymmetric Forked network to conditions that impair the MT network.

The timing and distribution of a polarized MT network during mid-oogenesis and the localization of the asymmetrical Forked-marked filamentous network are highly similar. Moreover, the the Forked network is dependent on polarized MTs, as revealed using *par-1* and *grk* mutants. These observations, together with the fact that Forked is associated with ncMTOC, support the use of GFP-Forked as a novel genetic reporter to study *Drosophila* oocyte polarity.

## MATERIALS AND METHODS

### Drosophila stocks

The following mutant and transgenic flies were used (See FlyBase for reference): *f*^36a^ (Hoover et al., 1993), *grk^2B 6^* (Neuman-Silberberg and Schupbach, 1993), *grk^ΔFRT^* (Lan et al., 2010), *par-1^W3^* (Shulman et al., 2000), *par-1 ^6323^* (Shulman et al., 2000), mCherry-Patronin, Shot-YFP (Nashchekin et al., 2016), Jupier^G00147^ (FBrf0161605) and Tau-GFP (Doerflinger et al., 2003). *jvl*^1^, *jvl*^f00590^ *shot-RNAi* (#41858), *Df(1)BSC584* (#25418) and *ik2-RNAi* (#35266) were obtained from the Bloomington Stock Center. Germline expression was performed with P{*matα4*-GAL4-VP16} V37 (hereafter referred to as *alpha-tub*), also obtained from the Bloomington Stock Center. Ovaries of the following genotypes were analyzed: (1) *alpha-tub>* GFP-Forked, (2) *alpha-tub>* mCherry-Forked, (3) *grk^2B6^*/*grk^ΔFRT^*; *alpha-tub>* GFP-Forked, (4) *par-1^w3^*/*par-1^6323^; alpha-tub>* GFP-Forked*,* (5) *alpha-tub>* mCherry-Forked*/shot-RNAi,* (6*) alpha-tub-2>* mCherry-Forked*/Shot-YFP* and (7) *alpha-tub>* mCherry-Patronin; GFP- Forked.

### Transgenic flies

The *forked* gene contains nine alternative splice forms. In this study, we mainly used the *forked-C* isoform, which was studied before (Hoover et al., 1993; Petersen et al., 1994). However, since it was shown that extra copies of *forked* affected bristle morphology (Petersen et al., 1994), we amplified a truncated form of the *forked-C* isoform from pupal cDNA using the following forward primer: 5′- GGAGTTCGTGACCGCCGCCGGGATCACTCTCGGCATGGACGAGCTGTACAAGTCTAGAATGACCACAAGTCTGACCTC-3′ and reverse primer**-** 5′ TATCAAGCTCCTCGAGTTAACGTTACGTTAACGTTAACGTTCGAGGTCGACTCTAGATCAGAGCAGCTTGGCTTTC-3′.

To make N-terminal GFP- or mCherry-Forked fusions using plasmids pUASp-GFP::Forked and pUASp-mCherry::Forked, DNA for GFP and mCherry was cloned into plasmid pUASp using the *Kpn*I and *Xba*I restriction sites by Gibson assembly (hereafter, the constructs are designated as plasmids pUASp-GFP and pUASp-mCherry). To make an N-terminal GFP-Forked fusion using plasmid pUASt-GFP::Forked, GFP was cloned into plasmid pUASt using the *Kpn*I and *Xba*I restriction sites (hereafter, the construct is designated as plasmid pUASt-GFP). *forked-C* was then cloned into plasmids pUASp-GFP, pUASt-GFP and pUASp-mCherry using the *Xba*I restriction sites. P-element-mediated germline transformation of these constructs was carried out by BestGene.

For generating *forked* knockout flies, appropriate guide RNA sequences were identified (Table S3) at http://tools.flycrispr.molbio.wisc.edu/targetFinder/ and cloned into plasmid pU6-BbsI-chiRNA. Then, 1 Kb sequences stretches upstream and downstream of *forked* were cloned into the donor pHD-DsRed-attP vector. Finally, injection of both vectors and fly screening was carried out by BestGene.

### Fertility assay

Three virgin females of the respective genotypes were mated with two wild-type males in a vial containing yeast for 2 days. Matings were performed in triplicate for each genotype. The flies were transferred to new vials containing fresh yeast for 1 day to lay eggs. The flies were discarded and the progeny resulting from the eggs after 10 days at 25°C were collected and counted. From each vial, the number of progeny per female, and the average number and standard deviation of progeny per genotype were calculated. Finally, a percentage of relative fertility was calculated (Spracklen et al., 2014).

### Drug treatment

Flies that had been starved for 2 h were fed 200 μg/ml colchicine for 16 h. The ovaries were dissected in PBS and fixed in 4% paraformaldehyde (PFA), followed by Alexa Fluor 568 phalloidin staining for actin.

### Immunohistochemistry

Ovaries were dissected in PBS and fixed for 10 min in 4% PFA. For actin staining, Oregon green 488 and Alexa Fluor 568 phalloidin dyes were used (Life Technologies). For antibody staining, anti-grk (1:50) and anti-staufen (1:500) antibodies were used. All images were taken on an Olympus FV1000 laser-scanning confocal microscope.

### Colocalization analysis

Fiji ImageJ software was used for colocalization analysis. First, the merged images were separated into two channels using the split channel option. The split images were analyzed and a colocalization pixel map was generated using the colocalization plug-in.

## Supplementary Material

Supplementary information
