# Supplementary material for: GFP-Forked, a genetic reporter for studying Drosophila oocyte polarity
Source: Biol Open. 2018 Dec 31;8(1):bio039552. doi: 10.1242/bio.039552 (PMC6361205; doi:10.1242/bio.039552)
Supplement: Supplementary information [file biolopen-8-039552-s1.pdf]

Table S1: forked null allele (CRISPR KO) has no effect on female fertility.

**WT**					
Vials no	Number of progeny from a 24h egg collection	Number of progeny per female	Avg	STD	% relative fertility
1	59	55	55	5	100
2	65	50			
3	62	60			
***forked* KO/*forked* KO**					
Vials no	Number of progeny from a 24h egg collection	Number of progeny per female	Avg	STD	% relative fertility
1	59	46	46	6	83.6
2	62	40			
3	50	52			

Table S2: Overexpression of Forked in the germline has no effect on female fertility.

***alpha-tub*-GAL4-VP16/*alpha-tub*- GAL4-VP16**					
Vials no	Number of progeny from a 24h egg collection	Number of progeny per female	Avg	STD	% relative fertility
1	71	23	23	3.05505	100
2	63	21			
3	81	27			
***alpha-tub*> GFP-Forked / *alpha-tub*> GFP-Forked**					
Vials no	Number of progeny from a 24h egg collection	Number of progeny per female	Avg	STD	% relative fertility
1	39	13	19	7.21	82.6
2	51	17			
3	81	27			

Biology Open • Supplementary information

Table S3. Guide RNA sequences for forked knock out.

Forked Knock-Out	gRNA sequences
Site 1	CTTCGATCGAGAGCGGGAGAGGGC
	AAACGCCCTCTCCCGCTCTCGATC
Site 2	CTTCGGAGAACCATGTCGATCCCA
	AAACTGGGATCGACATGGTTCTCC

Biology Open • Supplementary information

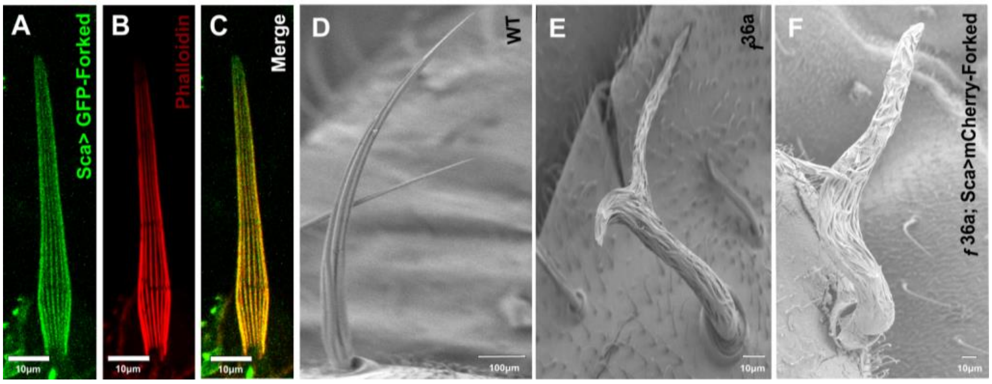

Figure S1: A truncated form of GFP-tagged Forked isoform C fails to rescue the *forked* mutant bristle phenotype.

Confocal Z-series projections of a 40 h APF bristle from WT fly expressing (A) *Sca* > GFP-Forked truncated form of isoform C stained with phalloidin for actin (red) (B). (C) Merged image. Scanning electron micrograph of an adult bristle from flies of (D) the wild type, (E) f^{36a}, showing severe morphological defects and (F) f^{36a}; *Sca* > UAS-mCherry-Forked truncated form of isoform C, showing defects similar to those seen in the f^{36a} mutant.

Biology Open • Supplementary information

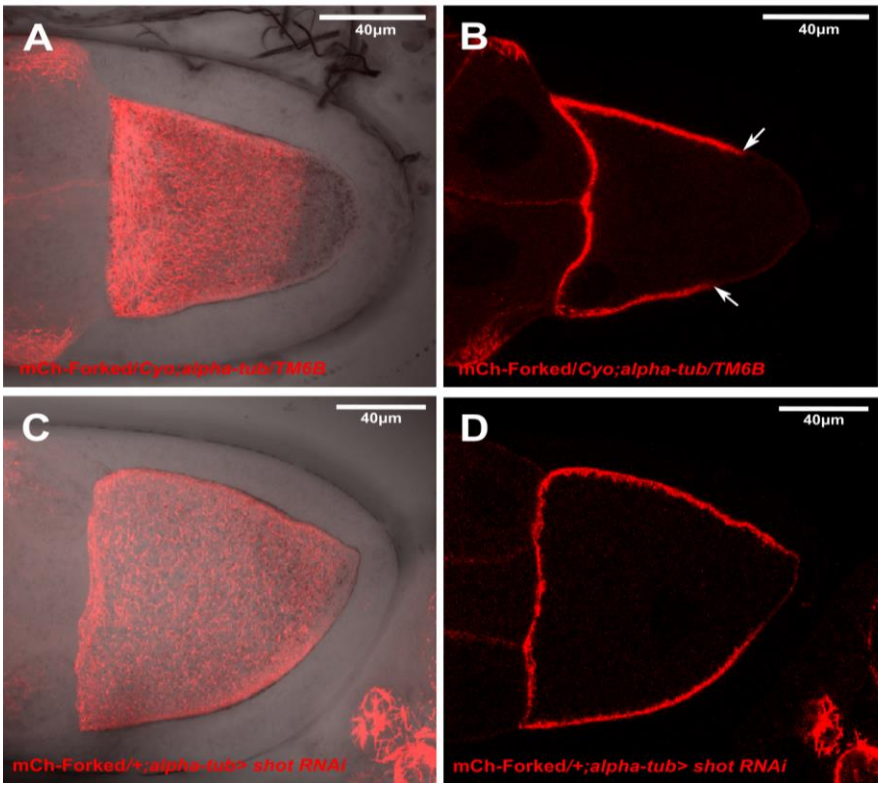

Figure S2: The asymmetric Forked network depends on Shot-Stop

Confocal Z-series projections merged with DIC of stage 10 (A) egg chambers from flies expressing *alpha-tub*> mCherry-Forked and (C) expressing both *Shot RNAi* and *alpha-tub*> mCherry-Forked. (B) and (D) are confocal slices from image (A) and (C), respectively. Arrows in (B) point towards the limit of the asymmetric network marked by mCherry-Forked.
